# Spleno-Mesenteric Venous Blood Flow Dynamics in Adult Patients with Chronic Portal Vein Thrombosis Analyzed by Sequential CT-Spleno- and Mesenterico-Portography

**DOI:** 10.3390/life15010129

**Published:** 2025-01-20

**Authors:** Alexandra Schlitt, Andrea Goetz, Christian Stroszczynski, Florian Zeman, Christina Hackl, Hans J. Schlitt, Ernst-Michael Jung, Wibke Uller, Simone Hammer

**Affiliations:** 1 Department of Radiology, University Medical Center Regensburg, 93053 Regensburg, Germanychristian.stros@ukr.de (C.S.); ernst-michael.jung@ukr.de (E.-M.J.); simone.hammer@ukr.de (S.H.); 2 Center for Clinical Studies, University Medical Center Regensburg, 93053 Regensburg, Germany; 3 Department of Surgery, University Medical Center Regensburg, 93053 Regensburg, Germany; christina.hackl@ukr.de (C.H.); hans.schlitt@ukr.de (H.J.S.); 4 Department of Diagnostic and Interventional Radiology, Medical Center, Faculty of Medicine, University of Freiburg, 79106 Freiburg, Germany; wibke.uller@uniklinik-freiburg.de

**Keywords:** hypertension (portal), portal vein thrombosis, radiology (interventional), hemodynamics, tomography (X-ray computed), therapy planning

## Abstract

Background: Portal vein thrombosis (PVT) leads to portal hypertension (PH) with its sequelae. Computed tomography spleno-mesenterico-portography (CT-SMPG) combines sequential CT spleno-portography and CT mesenterico-portography. CT-SMPG comprehensively illustrates the venous hemodynamic changes due to PH. Objective: To assess the effects of PV confluence thrombosis (PVCT) and liver cirrhosis on venous blood flow characteristics of patients with PVT. Method: CT-SMPG was performed in 21 patients with chronic PVT. CT-SMPG was compared to standard contrast-enhanced CT (CECT) and gastroscopy concerning the patency of splanchnic veins, varices and venous congestion. Results: PVCT had a significant effect on perfusion patterns: in patients without PVCT, esophageal varices (EV) and gastric varices were supplied by either the splenic vein (SV), the superior mesenteric vein (SMV), or both. In patients with PVCT, EV and gastric varices were mostly supplied by the SV (*p* = 0.021, *p* = 0.016). In patients without PVCT, small bowel varices were fed by both systems or the SMV, while in patients with PVCT they were fed by the SMV (*p* = 0.031). No statistically significant changes were detected regarding gastropathy, colorectal varices and small bowel congestion. Liver cirrhosis had no statistically relevant effect on hemodynamics. Conclusions: In CT-SMPG, patients with PVCT showed different venous hemodynamics to patients without PVCT, and this can serve as a basis for selecting therapy options.

## 1. Introduction

Portal vein thrombosis (PVT) is defined as thrombus formation within the portal vein (PV) trunk and the intrahepatic PV, sometimes even involving the superior mesenteric vein (SMV) and the splenic vein (SV). Formerly, PVT was classified as acute versus chronic but has recently been updated to acute symptomatic and non-acute symptomatic PVT [1]. Liver cirrhosis is the most common non-malignant cause for chronic PVT. Inherited or acquired prothrombotic states (e.g., polycythemia vera or factor V Leiden mutation) as well as tumor thrombosis are non-cirrhotic causes of PVT [2]. Also, recent research has shown that viral infections such as SARS-CoV-2 infections may lead to splanchnic vein thrombosis [3].

Portal hypertension (PH) increases with non-acute PVT and results in further hemodynamic changes: collateral pathways develop for bypassing the site of thrombosis. Depending on location, extension and stage of thrombosis, collateral circulation may be hepatofugal or hepatopetal [4]. The most common changes are development of portosystemic shunts, dilatation of veins resulting in varices, and venous congestion in the intestinal tract [5]. Symptoms of PH include ascites, splenomegaly and varices with or without bleeding [4]. Since PH can be clinically silent, acute gastric or esophageal bleeding may represent the first symptoms of PH [6].

To detect blood flow anomalies in the PV, different diagnostic methods can be used. According to the Baveno VII Consensus Doppler ultrasound, CT and MRI are the standard diagnostic methods for detecting PVT [7]; contrast-enhanced ultrasound (CEUS) can be used to further characterize PV anomalies [8]. Where Doppler ultrasound is used to diagnose PVT, additional contrast-enhanced computed tomography (CECT) or MR angiography is needed [7]. To determine whether or not PH is present, the gold standard is measuring the portal venous pressure gradient [7]. CECT and magnetic resonance imaging (MRI) are recommended to detect the extent of PV occlusion and involvement of additional vessels [6]. Endoscopy is used to detect esophageal, gastric and colorectal varices, gastropathy and small bowel congestion [7]. These techniques differentiation between patent and occluded vessels and the presence of varices but do not address prehepatic hemodynamic changes, feeding of varices and, consequently, prehepatic blood flow directions. Computed tomography (CT) during arterial portography proved capable of evaluating the portal venous system in a detailed way [9]. Moreover, this technique detects individual hemodynamic changes in children with PH [10] and was superior to standard cross-sectional imaging concerning confident assessment of the venous spleno-mesenterico-portal axis [11].

The therapy for PH in patients with PVT is not defined by the Baveno VII guidelines [7]. It is recommended to refer patients to an expert center for vascular interventional procedures. To determine an adequate therapy of symptoms, the pressure within the portal system has to be reduced. As transjugular intrahepatic portosystemic stent shunting (TIPSS) is usually not an option in patients with PVT, other options must be considered. Therapies may include splenic embolization, surgical portosystemic shunting and transvascular occlusion of varices. To decide on the best therapy for the individual, CT-SMPG may provide relevant information about the blood flow within the PV system and especially in varices [12].

The purpose of this study was to investigate hemodynamic changes using CT spleno-mesenterico portography (CT-SMPG) in adult patients with non-acute symptomatic PVT. Results of CT-SMPG were compared to standard CECT and endoscopy results. Furthermore, influence of (1) localization and extension of thrombosis and (2) cirrhosis on hemodynamic changes were examined using this technique.

## 2. Materials and Methods

This retrospective study was conducted according to the principles expressed in the Declaration of Helsinki and institutional review board approval was obtained (ethical approval number 20-1696-101). Inclusion criteria were CT-SMPGs performed in adult patients in our tertiary referral university center. All patients were previously diagnosed with PVT using ultrasound and additional cross-sectional imaging. The exclusion criterion was acute symptomatic PVT.

### 2.1. Technique

CT-SMPG is a combination of two methods: CT spleno-portography (CT-SPG) and CT mesenterico-portography (CT-MPG). For CT-SPG, the contrast medium is injected into the splenic artery (SA) to display the SV system and its drainage into the PV. For CT-MPG, the contrast medium is injected into the superior mesenteric artery (SMA) to display the SMV system and its drainage into the PV. CT-SMPGs were performed using a prospectively determined protocol for adult patients. Procedures were performed under local anesthesia. The SMA and SA were selectively catheterized using 4–5 French catheters introduced via a femoral access. Contrast medium (Imeron 300 or Accupaque 300) was selectively injected in the SMA and SA. Using a digital subtraction angiography (DSA), the time from the start of each injection until the PV or collateral vessels were contrasted was measured. Catheters were secured at the groin and patients were transferred to the CT suite. The CT-SPG scan was started after the angiographically predetermined time after application of 40 mL intravenous injection of Accupaque 350 into the SA catheter with a flow rate of 2.5 mL/s. For the CT-MPG scan, the same procedure was repeated with contrast injection into the SMA. Sequential CTs were performed using a helical 256 (2 × 128)-slice dual source CT scanner (Somatom Flash scanner; Siemens, Forchheim, Germany).

### 2.2. Imaging Analysis

Two experienced radiologists, blinded to patients’ history and symptoms, evaluated CT-MPG and CT-SPG in consensus in random order using a structured template and a dedicated picture archiving and communication system viewer (Syngo; Siemens Healthcare GmbH, Erlangen, Germany).

Patency of the SMV, the SV and the PV confluence were documented. The presence of varices and venous congestion were evaluated and their blood supply (SV or SMV system) was assessed. Splenorenal and abdominal wall shunts were noted. Definition of liver cirrhosis was surface and parenchymal nodularity, parenchymal heterogeneity and hypertrophy/atrophy of liver lobes.

### 2.3. Comparison CT-SMPG with CECT and Endoscopy

CECT images in the portal venous phase (within an interval of <12 months before and after CT-SMPG, without any surgical or interventional treatment of PH during this interval) were evaluated by the same two readers in consensus (blinded to patients’ data and results of CT-SMPG). Results of endoscopy performed within 12 months before or after the CT-SMPG by board-certified internists with several years of experience in endoscopy were recorded.

### 2.4. Statistical Analysis

Categorical variables were reported as frequency and percentage. Patients were further divided into (1) patients with PV thrombosis involving the PV confluence and patients without involvement of the PV confluence, and (2) patients with and without liver cirrhosis. Using cross tables, the effect of an open versus an occluded PV confluence and the effect of liver cirrhosis on the supply of the particular varices (SV, SMV or both) and venous congestions were compared. McNemar tests were used for statistical comparisons. A *p*-value < 0.05 was considered statistically significant. All analyses were performed using SPSS (version 29, IBM, Chicago, IL, USA).

## 3. Results

### 3.1. Study Cohort

**Figure 1:**
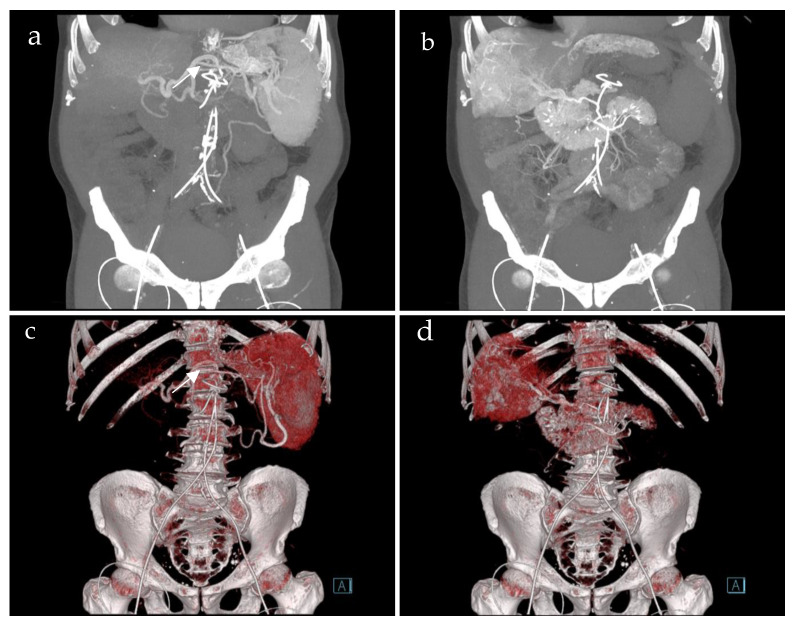
Coronal images of computed tomography spleno-mesenterico-portography of a 72-year old patient with portal hypertension showing the catheters in the splenic artery (**a**,**c**) (white arrow) and the superior mesenteric artery (**b**,**d**), both via the groin. Maximum intensity projection (**a**,**b**) and 3D models (**c**,**d**). Computed tomography splenoportography (**a**,**c**) detects the contrasted enlarged spleen, the splenic vein and extrahepatic portal vein. Computed tomography mesenterico-portography (**b**,**d**) shows the contrasted duodenum, liver and extrahepatic portal vein.

A total of 36 CT-SMPGs were performed in adult patients during an eight-year period. A total of 11 patients who suffered from acute PVT were excluded. Accordingly, 21 patients with chronic PVT who underwent CT-SMPG were included in this study (10 female, median age 47, 22–72 years). CT-SMPG indications were therapy planning of symptomatic PH with PVT in all patients. CT-SMPG was technically successful in all 21 cases. No procedure-related complications occurred. Figure 1 demonstrates an example of abdominal blood supply using CT-SMPG.

All 21 patients were diagnosed with PVT previously, using ultrasound and cross-sectional imaging on all patients (CECT 15, MRI two, both four). A total of 11 patients suffered from progressive gastrointestinal varices, while 10 patients suffered from severe gastrointestinal bleeding. In addition to the varices, four patients suffered from ascites resistant to treatment. Hepatic vein pressure gradient was measured in one patient. All other patients did not receive the invasive diagnostic modality since PH was already symptomatic. In five out of 21 patients, coagulopathies (JAK2-mutation, polycythemia vera, thalassemia, antiphospholipid syndrome) were detected as the reason for PVT.

### 3.2. Evaluation of Varices, Blood Flow and Portosystemic Shunting

All 21 patients showed abnormal perfusion patterns with the development of varices (n = 4), venous congestion (n = 1) or both (n = 16). Varices were supplied by the SV, the SMV or both systems: Esophageal varices (EV) (10 SV, two SMV, seven both), gastric varices (nine SV, one SMV, six both), gastropathy (six SV, two SMV, six both), small bowel varices (zero SV, seven SMV, one both), colorectal varices (zero SV, four SMV, two both), small bowel congestion (zero SV, 11 SMV, zero both).

Thirteen patients showed spontaneous splenorenal shunts supplied by the SV, while five patients showed splenorenal shunts supplied by both the SV and, in a retrograde manner, the SMV via the SV. Two patients showed abdominal wall shunts fed by the SV whereas one patient showed abdominal wall shunts fed by the SMV.

### 3.3. CT-SMPG Compared to CECT

**Figure 2:**
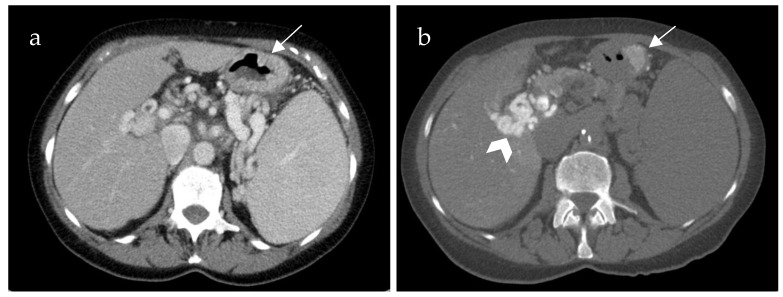
70-year-old female patient with portal hypertension due to chronic portal vein thrombosis. Axial images of contrast-enhanced computed tomography (CECT) (**a**) and computed tomography mesenterico-portography (CT-MPG) after contrast injection into the superior mesenteric artery (**b**). The CECT image shows diffuse contrast enhancement of the gastric wall (**a**) (white arrow). CT-MPG detects the partial hyperperfusion of the gastric wall (white arrow) and the cavernous transformation of the portal vein (white arrowhead (**b**)).

Additional CECT in the portal venous phase was available in 18 of 21 patients. Table 1 summarizes the diagnostic differences of CT-SMPG and CECT concerning detection of thrombosis of splanchnic veins (SMV, SV and confluence) and varices. CT-SMPG and CECT were quite similar in detecting PVCT, SMV and SV thrombosis whereas CT-SMPG was superior to CECT in detecting varices, gastropathy and venous congestion. Figure 2 shows an example of the same patient comparing CECT and CT-MPG.

### 3.4. CT-SMPG Compared to Endoscopy

Of 21 patients who underwent CT-SMPG, 11 patients underwent additional gastroscopy. Of these, both, gastroscopy and CT-SMPG detected EV in 10 cases. Gastric varices were noted by gastroscopy in five cases, and by CT-SMPG in nine cases. Gastropathy was detected in seven cases by gastroscopy, and in eight cases by CT-SMPG.

### 3.5. Hemodynamic Blood Flow Differences Between Isolated PVT and PVT Involving the Confluence

Table 2 shows the results of hemodynamic differences.

**Figure 3:**
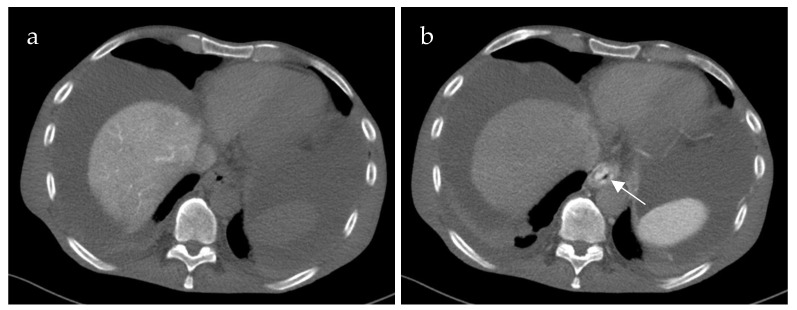
Computed tomography mesenterico-portography (**a**) and computed tomography splenoportography (**b**) of a 58-year-old patient with portal hypertension, portal vein thrombosis and portal vein confluence thrombosis. Esophageal varices (EV) are detected after contrast injection into the splenic artery ((**b**), white arrow). The EV do not show contrast enhancement after contrast injection into the superior mesenteric artery.

**Figure 4:**
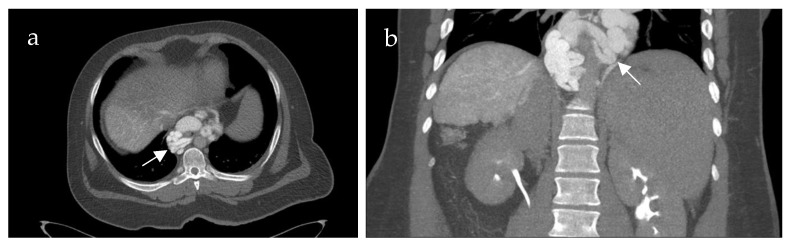
Computed tomography mesenterico-portography of a 24-year-old male patient with portal hypertension and portal vein thrombosis involving the confluence. Axial (**a**) and coronal (**b**) image after contrast injection into the superior mesenteric artery shows large contrasted periesophageal varices (white arrow) fed by the mesenteric system.

**Figure 5:**
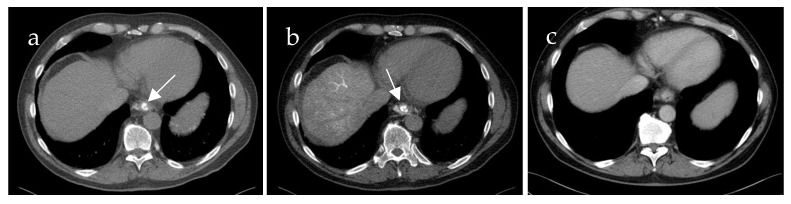
Patient of 61 years of age with portal hypertension and isolated portal vein thrombosis without involvement of the confluence. The computed tomography spleno-portography (**a**) shows contrasted esophageal varices (EV). The computed tomography mesenterico-portography (**b**) also shows contrasted EV. The EV are therefore fed by both the superior mesenteric vein and the splenic vein (white arrows). Contrast-enhanced computed tomography (**c**) after intravenous injection of contrast medium shows low contrast of all tissues and vessels. It does not reveal clearly distinguishable varices in the esophagus.

Inflow into esophageal, gastric and small bowel varices differed significantly between patients with PVCT (*p* = 0.021, *p* = 0.016 and *p* = 0.031, respectively) compared to patients without PVCT. In patients with PVCT, esophageal varices were fed solely by the SV in 75% (n = 9, example shown in Figure 3), solely by the mesenteric vein system in 8% (n = 1) and by both systems in 17% (n = 2, example of this rare case shown in Figure 4). Patients without PVCT showed a more equal distribution of inflow hemodynamics: esophageal varices were fed by solely the SV system in 22% (n = 2), by solely the SMV system in 22% (n = 2) and by both systems in 56% (n = 5, example of this is shown in Figure 5). In patients without PVCT, gastric varices were fed by the SV (22%, n = 2), by the SMV (11%, n = 1) or both (22%, n = 2). In contrast, 59% of the patients with PVCT (n = 7) showed gastric varices which were fed only by the SV. In 33% of cases (n = 4), both the SV and SMV drained into gastric varices. No inflow into gastric varices solely from the SMV was detected. These results were statistically significant (*p* = 0.016).

The inflow into small bowel varices differed significantly between patients with and without PVCT (*p* = 1.000 and *p* = 0.031). In one patient with an open PV confluence, the small bowel varices were fed by both systems (11%). Another patient with an open PV confluence showed small bowel varices fed only by the SMV. All other patients with PVCT showed small bowel varices and were all (55%, n = 6) fed exclusively by the SMV. Hence, small bowel varices were never fed only by the SV.

Small bowel congestion was detected in eight patients (73%) with PVCT and in three patients (33%) without PVCT. In all cases—whether open or occluded confluence—small bowel congestions were only fed by the SMV, never by the SV (*p* = 0.250 and *p* = 0.008).

Inflow into gastropathy and colorectal varices did not show significant differences in patients with and without PVCT (gastropathy *p* = 1.000 and *p* = 0.289, colorectal varices *p* = 1.000 and *p* = 0.25).

### 3.6. Influence of Liver Cirrhosis on Hemodynamic Changes in Patients with PVT

Table 3 shows the results of hemodynamic changes in patients with PVT and liver cirrhosis.

Liver cirrhosis did not influence the supply of varices (via SMV or SV). There were no statistically significant differences in the hemodynamics of EV (*p* = 0.219 and *p* = 0.219), gastric varices (*p* = 0.125 and *p* = 0.219), gastropathy (*p* = 0.219 and *p* = 1.0), small bowel varices (*p* = 0.250 and *p* = 0.125), small bowel congestion (*p* = 0.031 and *p* = 0.063) and colorectal varices (*p* = 0.125 and *p* = 1.000).

Etiologies of liver cirrhosis were spread between ethyl toxic, infectious, alpha-1-antitrypsin deficiency, primary sclerosing cholangitis, autoimmune hepatitis and idiopathic. Six patients were categorized Child Pugh A, six patients were categorized Child Pugh B, and one patient was categorized Child Pugh C. Albumin was below standard value in six patients, while the other cirrhosis patients had normal albumin levels.

### 3.7. Therapy Based on CT-SMPG Findings

Of all 21 patients who underwent CT-SMPG, 13 patients underwent surgical or radiologic interventional therapy after interdisciplinary discussion. Seven patients were treated using splenic embolization, three patients received a surgical porto-systemic shunt, one patient received both a surgical porto-systemic shunt and a splenic artery ligation, and two patients were treated using trans-splenic coiling/sclerosing of esophageal and/or gastric varices. All interventions/operations were primarily successful. Complications were noted in three patients (postembolization infection), which were treated successfully. All 13 patients could be discharged. Ten patients returned for follow-up, and three patients continued their follow-up in hospitals closer to their home town. In all, symptom reduction was noted using endoscopy (varices) and ultrasound (ascites) in 10 follow-up patients. Follow-up duration ranged from 2 months to 3 years and 3 months.

## 4. Discussion

CT-SMPG is a new approach for detection of hemodynamic changes in patients with (primarily prehepatic) PH used to identify potential therapeutic options. Initially, CT-SMPG was used to detect liver lesions but the sequential, selective contrasting of splanchnic vessels has been shown to have additional benefits in detecting changes in blood flow, development of varices, venous congestion and portosystemic shunts. Recent studies showed that this technique identifies hemodynamic changes in children with PH [10,11].

In our study, CT-SMPG was compared to CECT and endoscopy to ensure diagnostic validity. The results show a more effective assessment of the patency of splanchnic vessels using CT-SMPG than CECT. Furthermore, the CT-SMPG has proved to be more sensitive in the assessment of varices and venous congestions. In comparison to CECT, CT-SMPG benefits from the direct injection of contrast medium into either the SMA or the SA. Less dilution of the contrast medium by blood and the absence of contrasted surrounding tissue and arteries result in higher contrast between relevant venous structures and the surrounding structures. In this study, CT-SMPG also showed a more reliable detection of gastric varices (11/14 in CT-SMPG, 7/14 in gastroscopy) and gastropathy (8/14 in both CT-SMPG and gastroscopy) compared to gastroscopy, and only failed to detect EV I° in one patient. This ties in with the results of Sanada et al., who showed that CT-SMPG might even detect small varices not yet detectable in gastroscopy in children post-liver transplant [13]. Overall, the comparison between CT-SMPG and CECT showed that CT-SMPG is not inferior but rather equal, in some cases even superior, to the standard diagnostic methods in detecting varices and venous congestions due to higher contrast of relevant structures. Furthermore, CT-SMPG is an imaging modality that adds much more information than endoscopy alone.

Another aim of this study was to evaluate different possible influences on venous hemodynamics. In our study, the effect of PVCT and liver cirrhosis were examined as they could act as potential markers of hemodynamic changes. Concerning small bowel congestion, hemodynamics were similar between patients with PVT involving the confluence or not. The results show that small bowel congestion is fed by the SMV in all cases—whether open or occluded PV confluence. However, patients with PVCT develop a small bowel congestion more often than patients without PVCT. This may be due to the fact that high pressure within the SMV cannot be distributed over both systems (SMV and SV) but builds up in isolation in the SMV. Therefore, patients with an occluded PV confluence may benefit from closer monitoring regarding potential small bowel bleeding.

Though one could argue that liver cirrhosis could have an effect on the development of hemodynamic changes due to slow changes of portal pressure, CT-SMPG showed that there is no difference to patients without liver cirrhosis. This result could nevertheless be biased due to the fact that all included patients (with or without liver cirrhosis) also had a PVT and therefore an equal prehepatic cause for PH. Further research concerning the hemodynamic changes in patients without PVT with and without liver cirrhosis is required for a definite judgement of this matter.

In theory, the effectiveness of the different treatment options for PVT depends on whether the treated vessel or organ actually supplies the blood for the varices/venous congestions. For example, a splenomegaly, which is fed by only the SV, would profit from a splenic embolization or a distal spleno-renal-E/S-shunt (Warren shunt). On the other hand, a splenomegaly, which is fed by the SMV, would probably profit more from a mesocaval shunt. This study showed that supply of varices by the SMV would only be possible with an open PV confluence. So, especially in cases of PVT with open confluence, the CT-SMPG contributes relevant information for therapy planning. This study further detected that upper gastric varices are more likely to be fed by the SV rather than the SMV in patients with PVCT compared to patients with isolated PVT. There are, however, exceptions and, in these rare cases, a CT-SMPG may be beneficial to detect the individual hemodynamics. With regard to therapeutic options, the identification of the blood supplying venous system (SV versus SMV) is essential. Different approaches to reduce the pressure within the portal system can be used in order to reduce gastrointestinal bleeding or production of ascites. These approaches include TIPSS, partial splenic embolization and surgical splenorenal or mesocaval shunting. The therapeutic decision is based on various criteria, such as the primary symptoms of the PH, the access to repetitive treatment and the individual’s anatomic and vascular situation [14]. According to the experience of our interdisciplinary team of interventional radiologists, surgeons and gastroenterologists, information about the main venous supply of varices is important for the planning of successful therapeutic interventions to decompress the SMV or SV system. A short overview of therapies of our patients based on CT-SMPG findings showed promising results. However, future studies are necessary to determine the actual outcome of CT-SMPG-based decisions on therapeutic options.

Risk evaluation is essential when deciding the right diagnostic method to examine the sequelae of PH on the portosplenomesenteric venous axis. CECT and CT-SMPG both require X-ray exposure of the patient and application of contrast medium, which may lead to allergic reactions, thyreotoxic crisis or kidney failure. Both CT-SMPG and endoscopy are invasive methods with certain intervention-associated risks. CT-SMPG may cause peri- or postinterventional bleeding or infection. Endoscopy may cause mucous membrane injury, intestinal bleeding or infection. When choosing to perform a CT-SMPG for comprehensive evaluation of varices/organ hyperperfusions in patients with PH, an endoscopy and CECT including their additional risks could be avoided.

Limitations of this study were the retrospective study design and the small study cohort. Often patients with symptomatic PH and liver cirrhosis do not require therapy planning using CT-SMPG. According to the guidelines of the American Association for the Study of Liver Diseases, the gold standard to treat a PH without PVT is a TIPSS [15]. Hence, CT-SMPG is required for therapy planning in patients who are not suitable for TIPSS, mostly due to anatomical problems such as PVT. These complex cases require an individualized therapeutic approach in a highly specialized center.

Overall, the study showed that hemodynamics in patients with PH due to chronic PVT vary. The patency of the PV confluence may result in a change of original hemodynamics. In particular, the main supply of upper abdominal varices and organ hyperperfusions may differ. CT-SMPG is able to detect significant differences of hemodynamic changes in patients with isolated PVT and PVT with additional thrombosis of the confluence, and is a useful tool in identifying individual hemodynamic changes in order to guide potentially therapeutic options in selected complicated cases.

## Figures and Tables

**Table 1:** Detection of thrombosis of different splanchnic vein regions, varices and portosystemic.

Thrombosis of Splanchnic Veins	Detected by CT-SMPG ^1^ and CECT ^2^	Detected by CT-SMPG	Detected by CECT
Confluence	9	1	0
SMV ^3^	7	1	1
SV ^4^	7	2	0
Varices			
Esophageal varices	14	3	0
Gastric varices	10	4	1
Gastropathy	2	10	0
Small bowel varices	0	8	1
Small bowel congestion	1	8	1

^1^ CT-SMPG—Computed tomography spleno-mesenterico-portography, ^2^ CECT—Contrast-enhanced computed tomography, ^3^ SMV—Superior mesenteric vein, ^4^ SV—Splenic vein.

**Table 2:** Hemodynamic changes of patients with isolated portal vein thrombosis and patients with portal vein thrombosis involving the confluence.

	Patients Without PVCT ^1^ n = 9				*p*-Value	Patients with PVCT n = 12				*p*-Value
	Sup. ^2^ by SV ^3^	Sup. by SMV ^4^	Sup. by SV and SMV	No Varices/VC ^5^		Sup. by SV	Sup. by SMV	Sup. by SV and SMV	No Varices/VC	
Esophageal varices	11%	11%	56%	22%	1.000	75%	8%	17%	0	0.021
Gastric varices	22%	11%	22%	44%	1.000	59%	0	33%	8%	0.016
Gastropathy	0	0	33%	67%	1.000	50%	17%	25%	8%	0.289
Small bowel varices	0	11%	11%	78%	1.000	0	55%	0	45%	0.031
Small bowel congestion	0	33%	0	67%	0.250	0	73%	0	27%	0.008
Colorectal varices	0	17%	33%	50%	1.000	0	27%	0	73%	0.250

^1^ PVCT—Portal vein confluence thrombosis, ^2^ Sup.—Supplied, ^3^ SV—Splenic vein, ^4^ SMV—Superior mesenteric vein, ^5^ VC—venous congestion.

**Table 3:** Hemodynamic changes in patients with portal vein thrombosis and with or without liver cirrhosis.

	Patients without Liver Cirrhosis n = 9				*p*-Value	Patients with Liver Cirrhosis n = 12				*p*-Value
	Sup. ^1^ by SV ^2^	Sup. by SMV ^3^	Sup. by SV and SMV	No Varices/VC ^4^		Sup. by SV	Sup. by SMV	Sup. by SV and SMV	No Varices/VC	
Esophageal varices	50%	10%	30%	10%	0.219	46%	9%	36%	9%	0.219
Gastric varices	40%	0	50%	10%	0.125	46%	9%	9%	36%	0.219
Gastropathy	50%	10%	20%	20%	0.219	9%	9%	36%	46%	1.000
Small bowel varices	0	33%	0	67%	0.250	0	36%	9%	55%	0.125
Small bowel congestion	0	67%	0	33%	0.031	0	46%	0	55%	0.063
Colorectal varices	0	44%	0	56%	0.125	0	0	25%	75%	1.000

^1^ Sup.—Supplied, ^2^ SV—Splenic vein, ^3^ SMV—Superior mesenteric vein, ^4^ VC—venous congestion.

## Data Availability

The raw data supporting the conclusions of this article will be made available by the authors on request.

## Abbreviations

The following abbreviations are used in this manuscript:

CT	Computed tomography
CECT	Contrast-enhanced computed tomography
CT-SMPG	Computed tomography spleno-mesenterico-portography
CT-MPG	Computed tomography mesenterico-portography
CT-SPG	Computed tomography spleno-portography
DSA	Digital subtraction angiography
EV	Esophageal varices
IMV	Inferior mesenteric vein
MIP	Maximum intensity projection
MRI	Magnetic resonance imaging
SV	Splenic vein
SA	Splenic artery
SMA	Superior mesenteric artery
SMV	Superior mesenteric vein
Sup.	Supplied
PH	Portal hypertension
PV	Portal vein
PVT	Portal vein thrombosis
PVCT	Portal vein confluence thrombosis
TIPSS	Transjugular intrahepatic portosystemic stent shunt
VC	Venous congestion
